# Long-Term Effects of Maternal Low-Protein Diet and Post-weaning High-Fat Feeding on Glucose Metabolism and Hypothalamic POMC Promoter Methylation in Offspring Mice

**DOI:** 10.3389/fnut.2021.657848

**Published:** 2021-08-16

**Authors:** Jia Zheng, Ling Zhang, Jiayi Liu, Yanli Li, Junqing Zhang

**Affiliations:** ^1^Department of Endocrinology, Peking University First Hospital, Beijing, China; ^2^Department of Endocrinology, The Second Affiliated Hospital of Guangzhou Medical University, Guangzhou, China

**Keywords:** glucose metabolism, hypothalamus, DNA methylation, offspring, maternal low-protein diet, post-weaning high-fat feeding

## Abstract

Substantial evidence indicated that maternal malnutrition could increase the susceptibility to obesity, insulin resistance, and type 2 diabetes in adulthood. It is increasingly apparent that the brain, especially the hypothalamus, plays a critical role in glucose homeostasis. However, little information is known about the mechanisms linking maternal protein restriction combined with post-weaning high-fat (HF) feeding with altered expression of brain neurotransmitters, and investigations into the epigenetic modifications of hypothalamus in offspring have not been fully elucidated. Our objective was to explore the effects of maternal protein restriction combined with post-weaning HF feeding on glucose metabolism and hypothalamic POMC methylation in male offspring mice. C57/BL6 mice were fed on either low-protein (LP) or normal chow (NC) diet throughout gestation and lactation. Then, the male offspring were randomly weaned to either NC or high-fat (HF) diet until 32 weeks of age. Gene expressions and DNA methylation of hypothalamic proopiomelanocortin (POMC) and melanocortin receptor 4 (MC4R) were determined in male offspring. The results showed that birth weights and body weights at weaning were both significantly lower in male offspring mice of the dams fed with a LP diet. Maternal protein restriction combined with post-weaning high-fat feeding, predisposes higher body weight, persistent glucose intolerance (from weaning to 32 weeks of age), hyperinsulinemia, and hyperleptinemia in male offspring mice. POMC and MC4R expressions were significantly increased in offspring mice fed with maternal LP and postnatal high-fat diet (*P* < 0.05). Furthermore, maternal protein restriction combined with post-weaning high-fat feeding induced hypomethylation of POMC promoter in the hypothalamus (*P* < 0.05) and POMC-specific methylation (%) was negatively correlated with the glucose response to a glucose load in male offspring mice (*r* = −0.42, *P* = 0.039). In conclusion, maternal LP diet combined with post-weaning high-fat feeding predisposed the male offspring to impaired glucose metabolism and hypothalamic POMC hypomethylation. These findings can advance our thinking about hypothalamic POMC gene methylation between maternal LP diet combined with post-weaning high-fat feeding and metabolic health in offspring.

## Introduction

Maternal malnutrition has been associated with the onset of metabolic diseases in adulthood, including obesity, insulin resistance, and diabetes (1–4). It has been proposed to result from unbalanced dietary patterns during pregnancy and after weaning. Numerous animal experiments, including our previous studies, have indicated that maternal low-protein (LP) diet combined with a post-weaning high-fat (HF) diet can significantly increase susceptibility to obesity, impaired glucose tolerance, and insulin resistance in offspring (1, 2, 5).

However, the mechanisms underlying maternal and postnatal unbalanced diets and metabolic diseases in adulthood have not been fully elucidated. It has been widely accepted that epigenetic modifications may be the underlying mechanisms of these effects, which may link such imbalanced nutrition with the risks of metabolic diseases (6–8). It is reported that most peripheral organs, including the liver, pancreas, skeletal muscle, and adipose tissue appeared to be imprinted by unbalanced nutrition, which can be associated with epigenetic modulation of key developmental gene expressions (9, 10). Hypermethylation of these CpG islands has a specific effect on repressing transcription, whereas hypomethylation of CpG islands is related to transcriptional activation. When a CpG island in the promoter region of a gene is methylated, expression of the gene is repressed and *vice versa*. Our previous study showed that maternal LP diet can program glucose metabolism and hepatic microRNA expressions in early life offspring (11).

Recently, it became increasingly apparent that the brain, especially the hypothalamus, is the control center for energy homeostasis (12). Anorexigenic neuropeptides, located in the mediobasal hypothalamus, such as proopiomelanocortin (POMC), and melanocortin-4 receptor (MC4R), mediate satiety and increase energy expenditure, thus lead to loss of weight (13–15). Several studies have focused on the effects of maternal over-nutrition on hypothalamic neuropeptides, with increased expressions of POMC and MC4R in offspring (16–18). However, little information is known about the mechanisms linking prenatal LP and postnatal HF diets with altered expression of brain neurotransmitters, and investigations into the epigenetic modification of hypothalamus in offspring are limited. Our objective was to determine the programming effects of maternal protein restriction combined with post-weaning HF feeding on mice offspring, including metabolic health, hypothalamic neuropeptide gene expressions, and hypothalamic POMC gene methylation.

## Materials and Methods

### Ethical Statement

All experimental procedures were performed in accordance with the Guide for the Care and Use of Laboratory Animals, and procedures were approved by the Peking University First Hospital Institutional Animal Care and Use Committee.

### Experimental Design and Animal Model

Female C57BL/6J mice were maintained under controlled conditions and randomly assigned to either a LP diet (8% protein) or normal chow (NC) diet (20% protein) during pregnancy and lactation, as we previously described (5). Nutritional composition of the diets is shown in Supplementary Table 1. In order to avoid nutritional bias among litters, litter size was standardized to six pups. At 3 weeks of age, offspring mice were weaned either an HF diet (HF: 58% kcal fat) or an NC diet. Thus, it generated four groups of offspring mice: NC–NC, LP–NC, NC–HF, LP–HF (*n* = 8–10/group) (Abbreviations denoted before and after the dash line were as dam and offspring diets, respectively).

Birth weight of newborn mice and body weight at weaning were measured. Weight gain and food intake in offspring mice were recorded periodically. All the offspring mice were anesthetized and sacrificed at 32 weeks of age. Schematic representation of the experimental feeding course was shown in Supplementary Figure 1. Blood samples were collected from the retrobulbar, intraorbital, capillary plexus in anesthetized mice, which were fasted 10-h. The hypothalamuses were dissected, snap frozen, and stored at −80°C for further analysis, as we previously described (19). In this study, we mainly focused on male offspring to prevent confounding factors related to the estrus cycle and hormone profile of female offspring. In addition, a sexually dimorphic manner has been reported in the maternal LP diet rodent model, which was not the concern of this study (20, 21).

### Intraperitoneal Glucose Tolerance Tests

Intraperitoneal glucose tolerance tests (ipGTTs) were performed as previously described (22). Mice were fasted overnight (12 h) and injected intraperitoneally with glucose (2 g/kg body weight). Blood glucose concentrations were determined using a glucometer (Contour TS, Bayer, Beijing, P. R. China) and blood from the tail at baseline and 30-, 60-, and 120-min after glucose injection. Area under the curve (AUC) was calculated using the trapezoid method to evaluate blood glucose response to the ipGTTs.

### Serum Hormone Measurements

Serum insulin was detected using the Mouse Ultrasensitive Insulin ELISA kit (ALPCO Diagnostics, Salem, NH, USA) and serum leptin was measured using the mouse ELISA kits (R&D Systems, Minneapolis, MN, USA), according to the instructions of the manufacturers. Each sample was measured in duplicate.

### RNA Extraction and RT-qPCR Analyses

RNA was extracted from hypothalamus using TRIzol reagent (Life Technologies Inc., Carlsbad, CA, USA) and 1 μg RNA was converted into cDNA by a reverse transcription procedure using the Power cDNA Synthesis kit (Promega BioSciences LLC, Sunnyvale, CA, USA), according to the protocol of the manufacturer. Then, cDNA was amplified using the appropriate primers and probes. The sequences of the primers are as follows: POMC: forward 5′-CGACAGGCAGGAGACTGAAC-3′, reverse 5′-CGCAGAGAAACGAGGGTTTG-3′; MC4R: forward 5′-TGAACTTCTGAGAGGCTGCG-3′, reverse 5′-TTCTCGGTTGACCAGTCTGC-3′; and β-actin: forward 5′-TGTTACCAACTGGGACGACA-3′, reverse 5′-GGGGTGTTGAAGGTCTCAAA-3′. Real-time PCR was performed and accurately measured using a standard TaqMan PCR kit protocol on an ABI prism Vii7 Sequence Detection System (ABI Prism® Vii7, Applied Biosystems, Life Technologies). The relative expression levels were calculated using the 2^−Δ*ΔCt*^ method after normalization to the expression of the β-actin housekeeping gene (23). All reactions were carried out with three biological replicates, and each analysis consisted of three technical replicates.

### POMC and MC4R Methylation Levels by Bisulfite Sequencing PCR

POMC and MC4R promoters methylation levels were examined by bisulfite sequencing PCR, as our previous study described (19). Precisely, genomic DNA was extracted from hypothalamus tissues in offspring mice, using an E.Z.N.A. Tissue DNA Kit (Omega Bio-tek, Norcross, GA, United States), and DNA samples were treated with sodium bisulfite, using EZ DNA Methylation Kit (Zymo Research, HiSS Diagnostics, Germany), according to the instructions of the manufacturer. POMC and MC4R promoter areas were amplified using the following primers: POMC: forward 5′-GATTGGTTTTTGGGGAGATTT-3′, reverse 5′-ATTTCAAAACCTTAAACAATTCCCT-3′; MC4R: forward 5′-TTTAAAATTTGGAAAGGAAAATTT-3′, reverse 5′-TACTAAAAACAAAATCAAAAACAAC-3′; and β-actin: forward 5′-TGTTACCAACTGGGACGACA-3′, reverse 5′-GGGGTGTTGAAGGTCTCAAA-3′. PCR amplification of genomic fragment of POMC and MC4R promoters was performed using BIOTAQ DNA Polymerase (Bioline USA Inc, Taunton, MA, United States). The PCR products were separated on 1.5% agarose gel followed by gel extraction with QIAquick Gel Extraction Kit (QIAGEN, Hilden, Germany) and cloned into PGEMT-easy vectors (Promega, Madison, WI, United States). PGEMT-easy vectors were multiplied using JM109 competent *Escherichia coli* cells using standard procedures and then purified from the bacteria with QIAprep Spin Miniprep Kit (QIAGEN). At least 20 positive bacterial clones were conducted in each sample and a minimum of 95% bisulfite conversion was included in subsequent analyses. Figure generation, sequence analysis, and quality control were performed using BiQ Analyzer software.

### Statistical Analysis

Data are presented as mean values ± SEM. Statistical analysis was conducted through analyses of variance (ANOVAs), with repeated measures where applicable. Bonferroni *post hoc* tests were performed to identify where statistically significant differences existed when ANOVAs were significant. Group differences in fasting blood glucose, serum hormone measurements, mRNA expression levels, and DNA methylation levels were analyzed by one-way ANOVA. Body weight and ipGTT were analyzed by two-way ANOVA followed by Bonferroni *post hoc* test. Statistical significance was reached at a *P* < 0.05.

## Results

### Effects of Maternal Protein Restriction and Post-weaning High-Fat Feeding on Birth Weight, Body Weight, and Food Intake in Offspring

**Figure 1:**
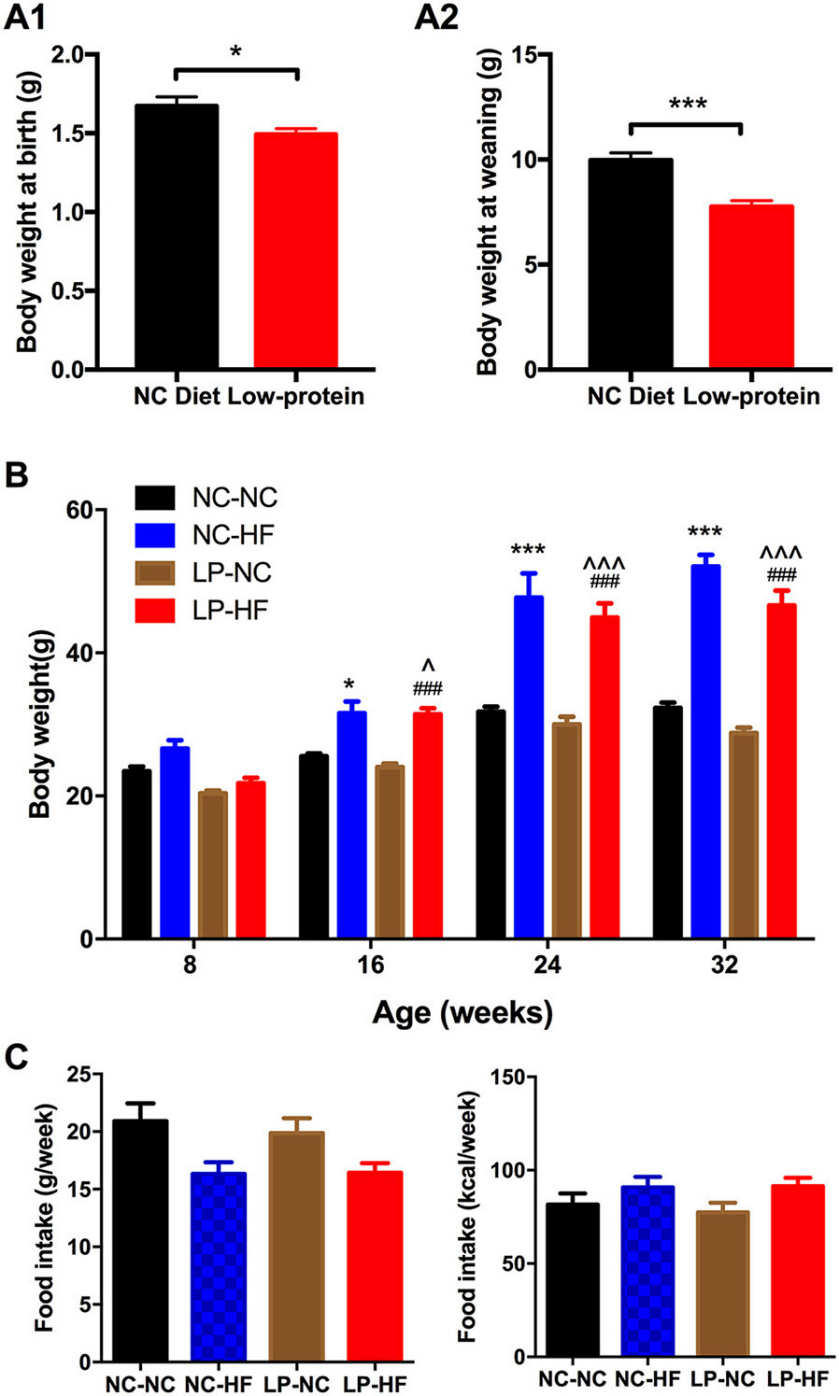
Effects of maternal protein restriction and post-weaning high-fat feeding on body weight and food intake in offspring. **(A)** Birth weight; **(B)** body weight at weaning; **(C)** body weight from 8 to 32 weeks of age. Data were represented as mean ± SEM (*n* = 6–8/group). *P < 0.05, ***P < 0.001 NC–HF *vs*. NC–NC group. ^###^*P* < 0.001 LP–HF *vs*. LP–NC group. ^∧^*P* < 0.05, ^∧∧∧^*P* < 0.001 LP–HF *vs*. NC–NC group. Diet abbreviations: NC, normal chow; LP, low protein; HF, high fat. Dam and pup diets denoted before and after the dash line, respectively.

Maternal LP diet during pregnancy and lactation induced lower birth weight in newborn mice (*P* < 0.05) (Figure 1A1). At weaning, body weight remained significantly decreased in mice offspring of dams fed with LP diet (*P* < 0.01) (Figure 1A2). At 8 weeks of age, no difference was found in body weight among the four groups. However, maternal LP diet combined with post-weaning HF-fed mice (LP–HF group) had increased body weight from 16-weeks of age until 32-weeks of age when mice were sacrificed, compared with both NC–NC and LP–NC groups (*P*-value as denoted) (Figure 1B). There was no difference in food consumption among offspring mice throughout the experiment (Figure 1C).

### Long-Term Effects of Maternal Protein Restriction and Post-weaning High-Fat Feeding on Fasted Blood Glucose and Glucose Tolerance in Offspring From 8 to 32 Weeks of Age

**Figure 2:**
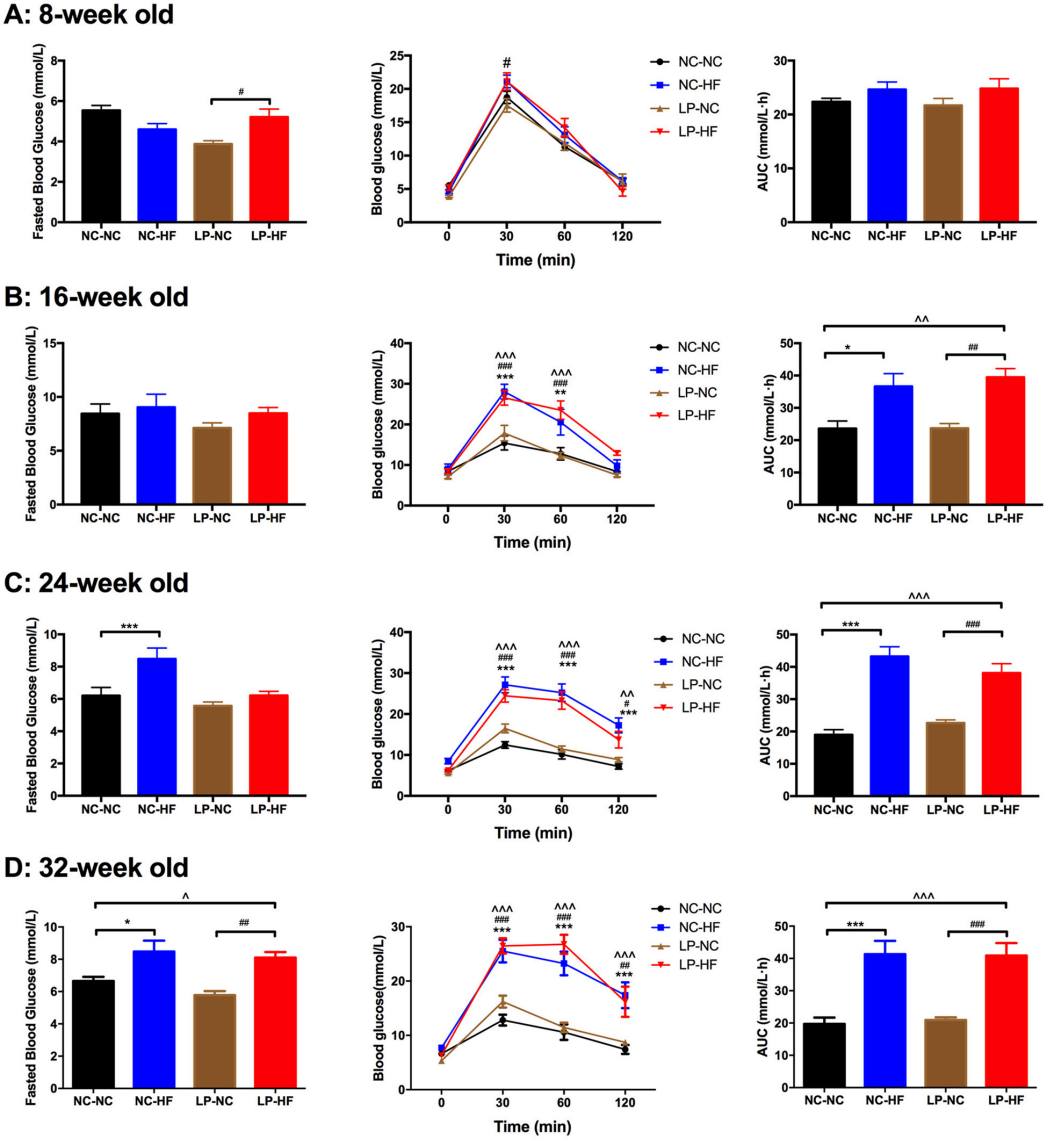
Long-term effects of maternal protein restriction and post-weaning high-fat feeding on fasted blood glucose and glucose tolerance in offspring from 8 to 32 weeks of age. **(A)** Glucose metabolism at 8 weeks of age; **(B)** glucose metabolism at 16 weeks of age; **(C)** glucose metabolism at 24 weeks of age; **(D)** glucose metabolism at 32 weeks of age. AUC: area under the curve of ipGTT; ipGTT: intraperitoneal glucose tolerance test. Data were represented as mean ± SEM (*n* = 6–8/group). *P < 0.05, **P < 0.01 NC–HF *vs*. NC–NC group, ***P < 0.001 NC–HF *vs*. NC–NC group. ^##^*P* < 0.01, ^###^*P* < 0.001 LP–HF *vs*. LP–NC group. ^∧∧^*P* < 0.01 LP–HF *vs*. NC–NC group, ^∧∧∧^*P* < 0.001 LP–HF *vs*. NC–NC group. Diet abbreviations: NC, normal chow; LP, low protein; HF, high fat. Dam and pup diets denoted before and after the dash line, respectively.

As shown in Figure 2 and Supplementary Figure 2, maternal protein restriction combined with post-weaning HF feeding (LP–HF) had impaired glucose tolerance from 8 weeks of age. At 8 weeks of age, fasted blood glucose concentration (*P* < 0.05) and blood glucose levels of the male offspring in the LP–HF group were significantly higher at 30 min (*P* < 0.05) after intraperitoneal glucose administration. However, there is no difference in AUC among the four groups. Then the glucose metabolism disturbance was exacerbated in the offspring of LP–HF group during the period from 8 to 32 weeks. At 32 weeks of age, fasted blood glucose concentration was significantly increased in offspring mice exposed to maternal protein restriction combined with post-weaning HF feeding, compared with NC–NC (*P* < 0.05) and LP–NC (*P* < 0.001) groups, respectively. The blood glucose levels of the male offspring in the NC–HF and LP–HF groups were significantly higher at 30 min (*P* < 0.001), 60 min (*P* < 0.001), and 120 min (*P* < 0.001) after intraperitoneal glucose administration, compared with those of the NC–NC offspring. Consistently, the AUC of ipGTT was significantly greater in NC–HF and LP–HF than NC–NC offspring (*P* < 0.001). Thus, it indicates that maternal protein restriction combined with post-weaning HF feeding, predisposes persistent glucose intolerance in offspring mice from weaning to 32 weeks of age.

### Maternal Protein Restriction and Post-weaning High-Fat Feeding Resulted in Hyperinsulinemia and Hyperleptinemia in Offspring Mice

**Figure 3:**
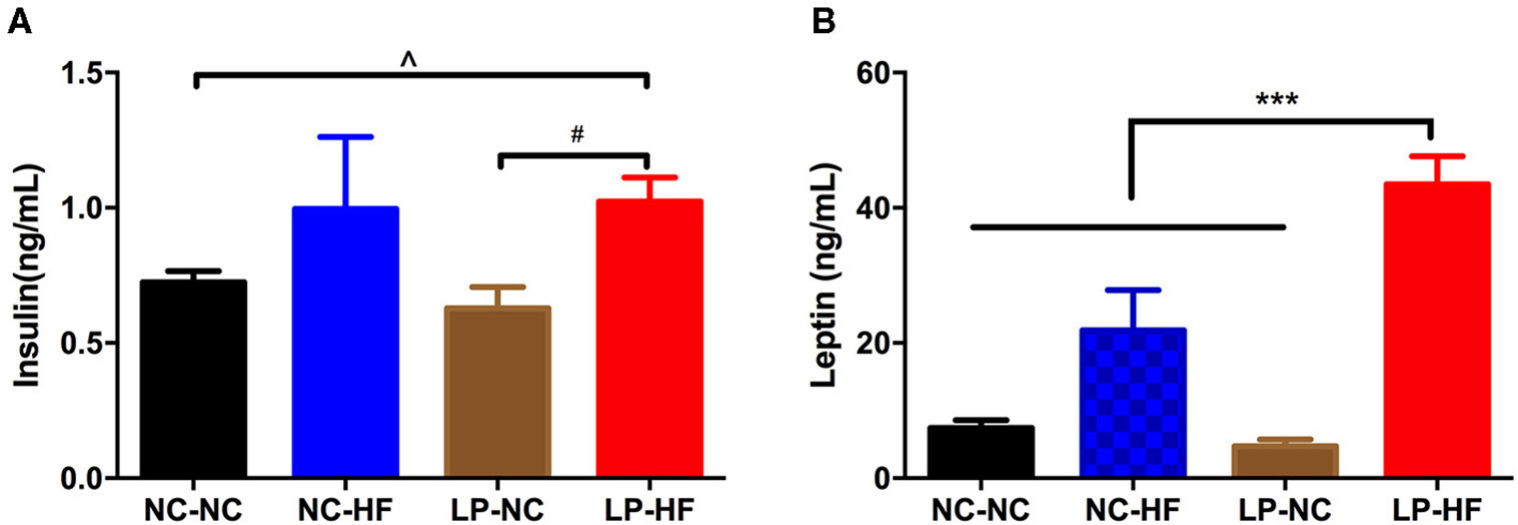
Maternal protein restriction and post-weaning high-fat feeding predisposes offspring to hyperinsulinemia and hyperleptinemia. **(A)** Serum insulin level; **(B)** serum leptin level. Data were represented as mean ± SEM (*n* = 6–8/group). *P*-value is significant as denoted. Diet abbreviations: NC, normal chow; LP, low protein; HF, high fat. Dam and pup diets denoted before and after the dash line, respectively. ***P < 0.001 NC–HF *vs*. NC–NC group. ^#^*P* < 0.05 LP–HF *vs*. LP–NC group. ^∧^*P* < 0.05 LP–HF *vs*. NC–NC group.

Serum insulin concentration was significantly increased in offspring mice fed an HF diet whose mothers had been fed an LP diet, compared with NC–NC and LP–NC groups (both *P* < 0.05) (Figure 3A). We further detected serum leptin level in offspring mice. As a critical peripheral hormone, leptin can act on leptin receptors located in the arcuate nucleus of the hypothalamus to regulate appetite and energy homeostasis. Leptin levels were significantly higher in HF-fed offspring whose mothers had eaten the LP diet, compared with all the other offspring mice (*P* < 0.001) (Figure 3B).

### Maternal Protein Restriction and Post-weaning High-Fat Feeding Regulated Hypothalamic POMC and MC4R Expressions in Offspring

**Figure 4:**
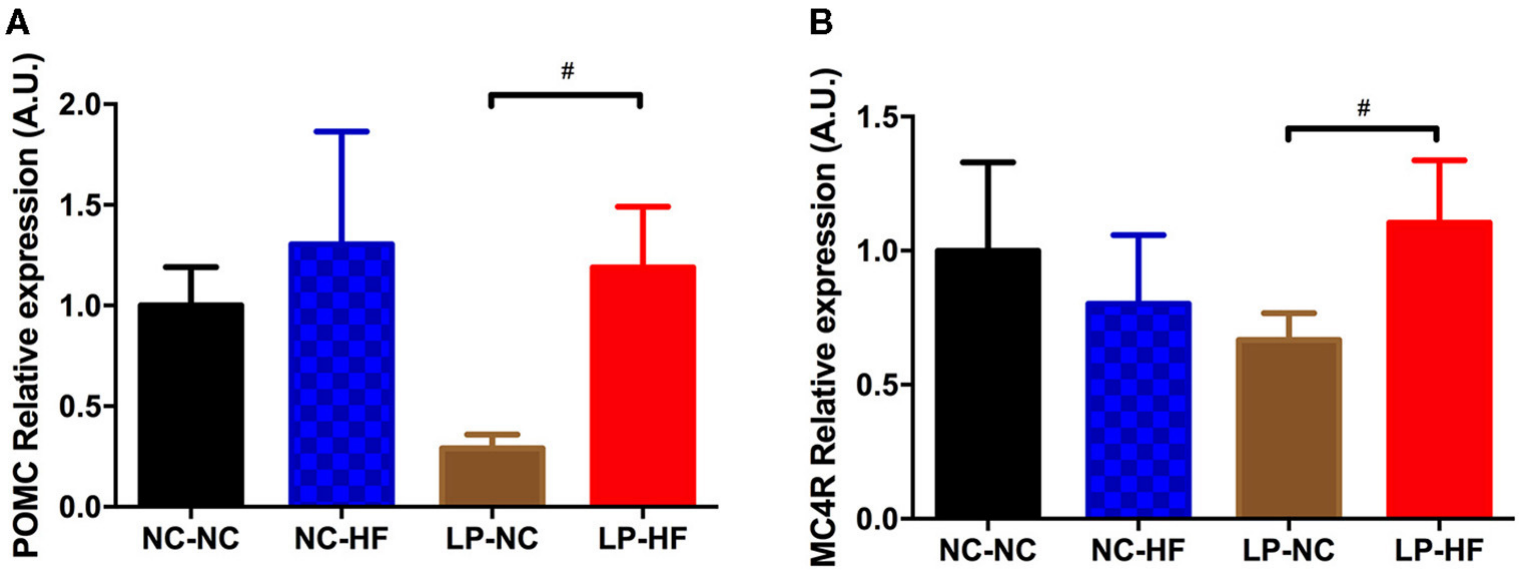
Effects of maternal protein restriction and post-weaning high-fat feeding on hypothalamic gene expressions in offspring. **(A)** POMC; **(B)** MC4R. Data were represented as mean ± SEM (*n* = 6–8/group). ^#^*P* < 0.05 LP–HF *vs*. LP–NC group. Diet abbreviations: NC, normal chow; LP, low protein; HF, high fat. Dam and pup diets denoted before and after the dash line, respectively.

To further assess the potential effects on the neuroendocrine control of body weight and energy homeostasis, we examined POMC and MC4R gene expression in the hypothalamus of the offspring. POMC and MC4R expressions were significantly increased in offspring mice exposed to maternal protein restriction combined with post-weaning HF feeding (both *P* < 0.05) (Figures 4A,B).

### Effects of Maternal Protein Restriction and Post-weaning High-Fat Feeding on POMC Methylation in Offspring

**Figure 5:**
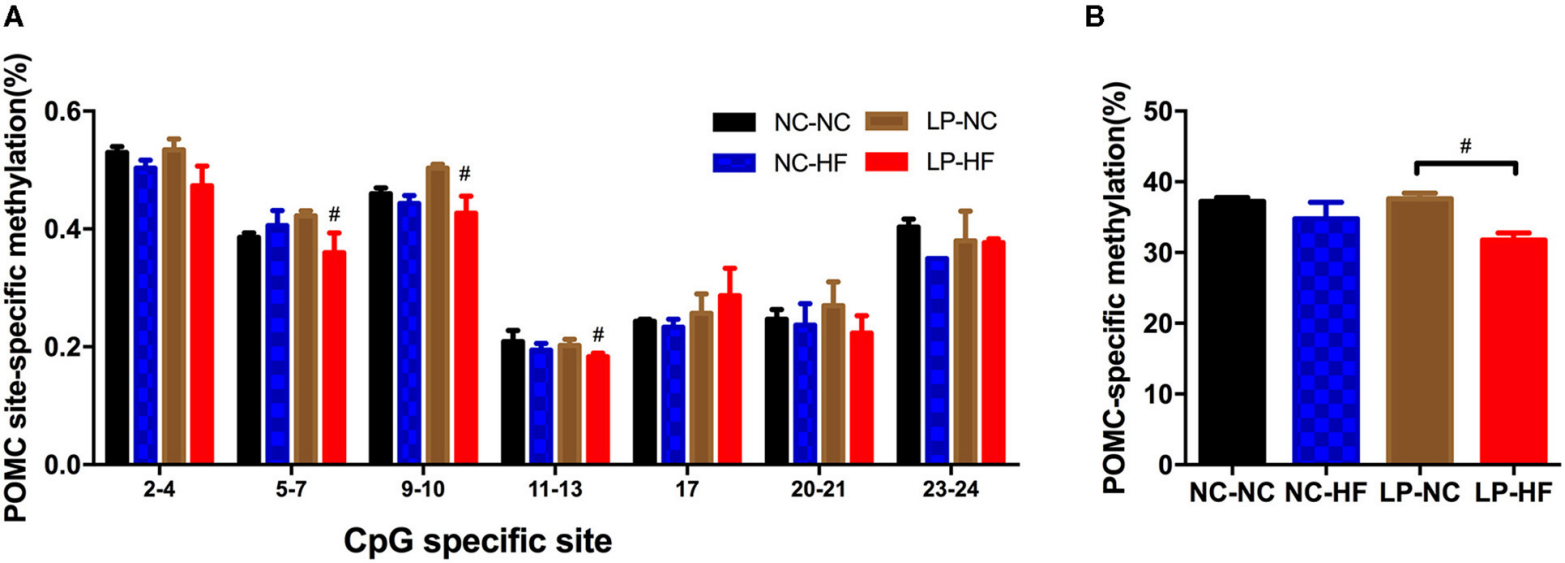
Effects of maternal protein restriction and post-weaning high-fat feeding on POMC methylation in offspring. **(A)** Methylation level (%) of specific CpG site in POMC gene promoter; **(B)** POMC-specific methylation (%). POMC site-specific methylation (%). Data were represented as mean ± SEM (*n* = 6–8/group). ^#^*P* < 0.05 LP–HF *vs*. LP–NC group. Diet abbreviations: NC, normal chow; LP, low protein; HF, high fat. Dam and pup diets denoted before and after the dash line, respectively.

Then, as one important epigenetic modification, DNA methylation levels of POMC and MC4R genes were further examined using MassARRAY EpiTYPER assays. We mainly concentrated on the CpG islands of POMC and MC4R gene promoters. We use online EMBOSS Cpgplot software to predict CpG island (http://www.ebi.ac.uk/Tools/seqstats/emboss_cpgplot/). According to the prediction results, POMC gene has one CpG island with 24 CpG sites; however, no CpG islands were predicted of MC4R gene promoter. For POMC, methylation level was decreased at the specific sites of 5–7, 9–10, and 11–13 (all *P* < 0.05) (Figure 5A). The average methylation level of POMC promoter was significantly decreased in the HF-fed offspring whose mothers were fed on an LP diet (*P* < 0.05) (Figure 5B).

### Correlation Between POMC-Specific Methylation and Glucose Metabolism in Offspring Mice

**Figure 6:**
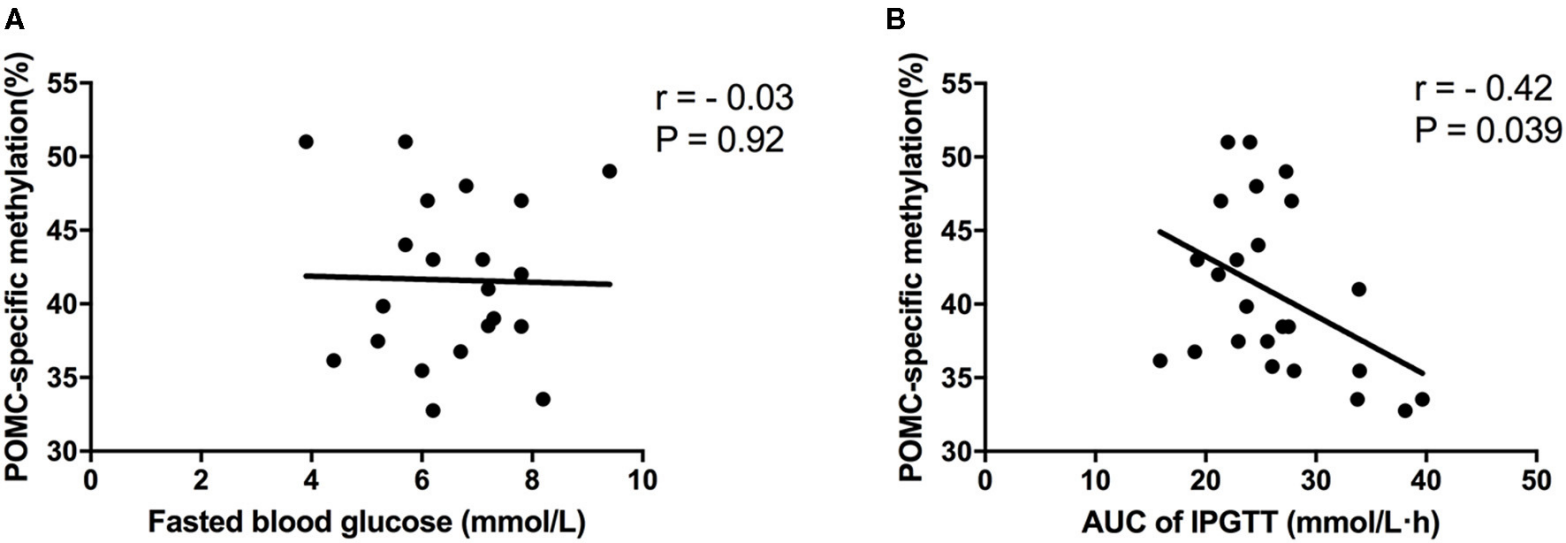
Correlation analyses between POMC methylation and glucose metabolism status. **(A)** POMC-specific methylation (%) and fasted blood glucose; **(B)** POMC-specific methylation (%) and AUC of ipGTT. Data were represented as mean ± SEM (*n* = 6–8/group). Diet abbreviations: NC, normal chow; LP, low protein; HF, high fat. Dam and pup diets denoted before and after the dash line, respectively.

To further evaluate whether differential POMC-specific methylation was responsible for impaired glucose metabolism due to maternal LP and postnatal HF diet in offspring mice, Spearman's correlation analyses were performed between POMC-specific methylation (%) and fasted blood glucose (10-h fasting before sacrifice) and AUC of ipGTT, respectively. No association was observed between POMC-specific methylation (%) and fasted blood glucose (*r* = −0.03, *P* = 0.92) (Figure 6A). Remarkably, it indicated that POMC-specific methylation (%) was negatively correlated with the glucose response to a glucose load in offspring mice (*r* = −0.42, *P* = 0.039) (Figure 6B).

## Discussion

It has been widely reported that prenatal LP and postnatal HF diets can induce the occurrence of long-term metabolic disorders in mammals, including obesity, glucose intolerance, and type 2 diabetes (24, 25). In our study, we showed that maternal LP diet induced adaptive changes, leading to obesity, impaired glucose tolerance, hyperinsulinemia, and hyperleptinemia when the mice were challenged with post-weaning high calorie intake. Consistently, previous studies showed that maternal and post-weaning imbalanced nutrition induced detrimental consequences on glucose homeostasis in adult life (1, 2, 26, 27). However, there is no difference in glucose tolerance between maternal LP diet and NC diet, both together with a post-weaning HF. It seems that the impaired glucose homeostasis mainly results from the HF feeding. Thus, a post-weaning HF diet results in the development of aberrant energy homeostasis and metabolic diseases in later life.

The central nervous system, mainly hypothalamus is the central control of whole body energy homeostasis (28). The arcuate nucleus in hypothalamus contains both anorexigenic and orexigenic neurons, which can counterbalance each other to regulate food intake and energy expenditure, and ultimately control body weight (29). POMC neuron, as the most-studied anorexigenic neuron in the arcuate nucleus, it can regulate energy homeostasis *via* MC4R in the paraventricular nucleus (30). It indicates that the peptides released from POMC neuron clearly play a role in reducing food consumption to control body weight. Increasing evidence has indicated that exposure to adverse maternal nutrition impairs hypothalamic development and function, which is plastic and sensitive to metabolic signals, potentially underpinning metabolic health in adult life (31). Our study indicated that both hypothalamic POMC and MC4R expressions significantly increased in offspring mice that were exposed to maternal protein restriction combined with post-weaning HF feeding. In addition, we found that serum insulin and leptin levels were significantly increased in HF-fed offspring whose mothers had eaten the LP diet. Consistent with our findings, Ikenasio-Thorpe et al. showed that prenatal under-nutrition (30% of *ad libitum* intake throughout gestation) and postnatal HF nutrition (45% kcal as fat) in Wistar rats exhibited increased food intake, obesity, and higher fat mass in offspring at 24 weeks of age, which correlated with hypothalamic POMC increment and circulating insulin and leptin level elevations (32).

Furthermore, we investigate the epigenetic status of POMC and MC4R gene in offspring mice. It indicated that hypothalamic POMC promoter methylation was significantly decreased in mice exposed to maternal LP diet and post-weaning HF feeding. However, no CpG island was detected of MC4R gene promoter, thus it restricted to evaluate methylation status of MC4R gene. It is widely acknowledged that hypomethylation of a certain gene can activate transcription and increase gene expression (33). In our study, we found that decreased methylation of POMC gene promoter was consistently related with increased gene expression in hypothalamus. Consistently, Stevens et al. showed a marked hypomethylation (62% decrease) of hypothalamic POMC promoter in the ovine fetus exposed to maternal diet intake restriction (34). It is widely accepted that epigenetic modifications mainly occur during early life development, which may continue throughout the lifespan (35). Our study indicated that maternal malnutrition and post-weaning HF diet can regulate epigenetic modifications in offspring mice at 32 weeks of age. This provides evidence that DNA methylation could play as a programming mechanism for hypothalamic POMC gene, which can regulate abnormal glucose metabolism through hypothalamic feeding center in later life. Of interest, our present study showed that POMC-specific methylation (%) was negatively correlated with the competence of glucose response to a glucose load. It indicated that POMC promoter methylation may be a critical epigenetic modification which can project to regulate food intake, body weight, and glucose metabolism in the next generation.

In conclusion, our study indicated that maternal LP diet combined with post-weaning HF feeding resulted in obesity, persistent glucose intolerance, hyperinsulinemia, and hyperleptinemia in offspring. We further found that POMC gene methylation status may be a potential mechanism for impaired glucose metabolism in offspring. These findings can advance our thinking about hypothalamic POMC gene methylation between maternal LP diet combined with post-weaning HF feeding and metabolic health in offspring.

## Data Availability Statement

The original contributions presented in the study are included in the article/Supplementary Material, further inquiries can be directed to the corresponding author/s.

## Ethics Statement

The animal study was reviewed and approved by Peking University First Hospital Institutional Animal Care and Use Committee.

## Author Contributions

JZha conceived and designed the experiments. JZhe, LZ, and JL carried out the experiments. JZhe and YL analyzed the data. All authors were involved in writing the paper and had final approval of the submitted and published versions.

## Conflict of Interest

The authors declare that the research was conducted in the absence of any commercial or financial relationships that could be construed as a potential conflict of interest.

## Publisher's Note

All claims expressed in this article are solely those of the authors and do not necessarily represent those of their affiliated organizations, or those of the publisher, the editors and the reviewers. Any product that may be evaluated in this article, or claim that may be made by its manufacturer, is not guaranteed or endorsed by the publisher.
