# Supplementary material for: Long-Term Effects of Maternal Low-Protein Diet and Post-weaning High-Fat Feeding on Glucose Metabolism and Hypothalamic POMC Promoter Methylation in Offspring Mice
Source: Front Nutr. 2021 Aug 16;8:657848. doi: 10.3389/fnut.2021.657848 (PMC8415226; doi:10.3389/fnut.2021.657848)
Supplement: Supplementary file 1 [file Table_1.DOCX]

**Supplementary Table 1. Nutritional composition of the diets fed to mice.**

| **Diets** | **normol chow (NC) diet** | | **low protein (LP) diet** | | **high-fat (HF) diet** | |
| --- | --- | --- | --- | --- | --- | --- |
| Diet components | g (%) | kcal (%) | g (%) | kcal (%) | g (%) | kcal (%) |
| Protein | 20% | 25% | 8% | 11% | 23% | 16.40% |
| Fat | 5% | 13% | 5% | 13% | 35.80% | 58% |
| Carbohydrates | 53% | 62% | 63% | 76% | 35.50% | 25.50% |
| Total |  | 100% |  | 100% |  | 100% |
| Total energy（kcal/g） |  | 3.45 |  | 3.33 |  | 5.56 |

**Supplementary Figure 1. Schematic representation of the experimental feeding course.**

Diet abbreviations: NC, Normal Chow diet; LP, Low protein diet; HF, High fat diet. Dam and pup diets denoted before and after the dash line, respectively.

**Supplementary Figure 2. Dynamic Effects of Diet on Fasted Blood Glucose and Glucose Tolerance in Offspring from 8 to 32 Weeks of Age. (A)** Fasted blood glucose in offspring mice from 8-week old to 32-week old; **(B)** Glucose tolerance in offspring mice from 8-week old to 32-week old. FBG: Fasted blood glucose, AUC: area under the curve of ipGTT, ipGTT: intraperitoneal glucose tolerance test. Data was represented as mean ± SEM (n =6-8/group). Diet abbreviations: NC, Normal chow; LP, Low-protein; HF, High-fat. Dam and pup diets denoted before and after the dash line, respectively.
