# Supplementary figures and images for: Long-Term Effects of Maternal Low-Protein Diet and Post-weaning High-Fat Feeding on Glucose Metabolism and Hypothalamic POMC Promoter Methylation in Offspring Mice
Source: Front Nutr. 2021 Aug 16;8:657848. doi: 10.3389/fnut.2021.657848 (PMC8415226; doi:10.3389/fnut.2021.657848)

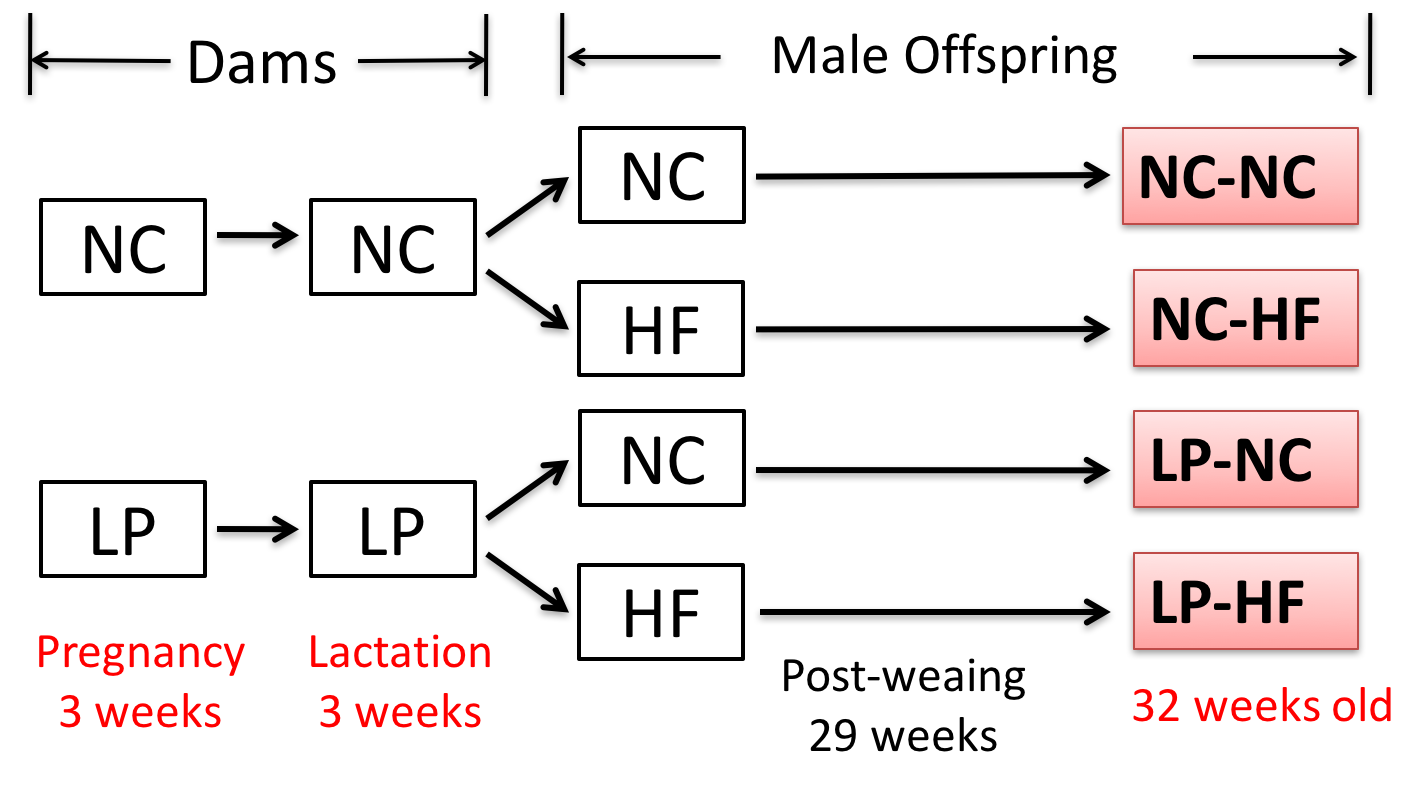

Supplement: Supplementary file 2 [file Image_1.PNG]

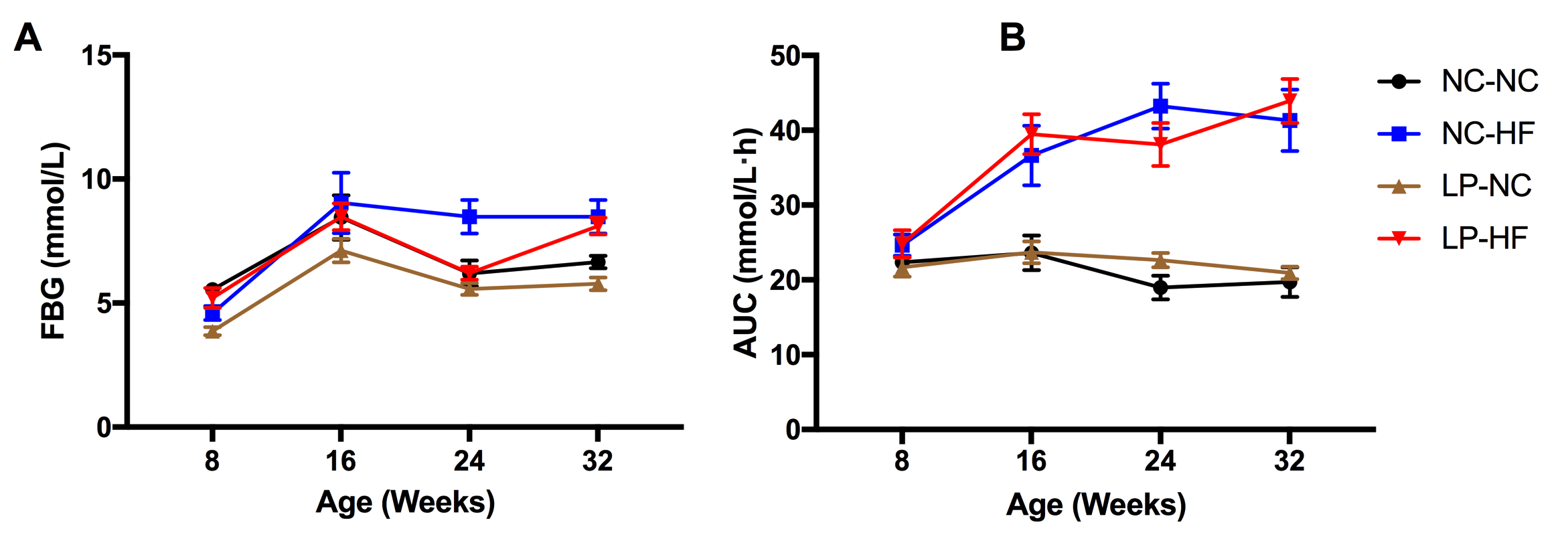

Supplement: Supplementary file 3 [file Image_2.TIFF]
